# Effect of Torrefaction Condensate on the Growth and Exopolysaccharide Production of *Chlamydomonas reinhardtii*

**DOI:** 10.3390/molecules30214313

**Published:** 2025-11-06

**Authors:** Salini Chandrasekharan Nair, Amal D. Premarathna, Anjana Hari, Christine Gardarin, Céline Laroche, Rando Tuvikene, Renu Geetha Bai, Timo Kikas

**Affiliations:** 1 Chair of Biosystems Engineering, Institute of Forestry and Engineering, Estonian University of Life Sciences, Kreutzwaldi 56, 51014 Tartu, Estonia; salini.chandrasekharan@emu.ee (S.C.N.); anjana.hari@emu.ee (A.H.); renu.bai@emu.ee (R.G.B.); 2 School of Natural Sciences and Health, Tallinn University, Narva mnt 29, 10120 Tallinn, Estonia; amald@tlu.ee (A.D.P.); rtu@akvaarium.com (R.T.); 3 Institut Pascal, Université Clermont Auvergne, UMR CNRS 6602, 63000 Clermont-Ferrand, France; christine.gardarin@uca.fr (C.G.); celine.laroche@uca.fr (C.L.)

**Keywords:** torrefaction, microalgae, amino acids, fatty acids, anti-oxidant assays, circular bio economy

## Abstract

Torrefaction, a mild thermochemical pretreatment process, generates the fuel-torrefied biomass along with non-condensable and condensable gases. The latter can be condensed to yield a dark, viscous liquid called torrefaction condensate (TC). In this study, we investigated the effect of TC on growth and exopolysaccharide (EPS) production by the green microalgae *Chlamydomonas reinhardtii*, a well-known model organism. Aspen wood pellets were torrefied at different temperatures, and the condensate formed at each temperature was analyzed. Based on the GC-MS analysis, 225 °C TC was selected and used for the cultivation of *C. reinhardtii*. Results show that at 2 mL/L and 2.5 mL/L concentrations, TC negatively impacts growth, EPS production, as well as the composition of amino acids, lipids, and fatty acids n of *C. reinhardtii*. However, *C. reinhardtii* gradually adapted to TC and attained the growth patterns comparable to the control, showing the resilience of the culture. The biochemical and antioxidant properties of the EPS showed significant differences to that of the control. Therefore, cultivating these microalgae in TC suggests a new microalgal biorefinery approach through the utilization of low-value TC for the production of value-added products, such as EPS.

## 1. Introduction

Over the past decades, the concept of circular bioeconomy has emerged as a promising solution to address the world’s energy demands and mitigate pollution. According to European bioeconomy policy, circular bioeconomy encompasses environmental, societal, and economic sustainability, aiming to maximize the value of biological resources while minimizing waste generation [1]. In circular bioeconomy, biomass plays an important role as the organic feedstock material for the production of fuel, food, and energy. Although biomass is considered a viable alternative to fossil fuels, it has some drawbacks, such as high moisture content, low calorific value, hydrophilic nature, and susceptibility to microbial degradation [2].

To address these challenges, thermochemical conversion processes such as combustion, pyrolysis, gasification, liquefaction, carbonization, and torrefaction have been introduced to produce heat energy or solid, liquid, and gas fuels [3,4]. In case of torrefaction, solid fuels are produced. Torrefaction particularly improves the fuel quality of biomass [5]. In torrefaction, biomass is treated in an oxygen-free environment at temperatures between 200 °C and 300 °C to improve the heating value and hydrophobicity [6]. Torrefied biomass exhibits properties similar to coal, making it a suitable substitute [7].

Apart from torrefied biomass, certain condensable and non-condensable gases are produced during torrefaction [8]. These gaseous byproducts primarily consist of carbon monoxide, carbon dioxide, and other volatile compounds. The condensable gases can be condensed in some reactor configurations to form a torrefaction condensate (TC), which consists of water, acetic acid, other organic acids, furfurals, furans, and phenols [9]. The wide distribution of molecular weight and polarity of the TC components make it difficult to separate and valorize these compounds [10]. Furthermore, the high concentration of acids and phenolics limit the application of TC and makes it difficult for microbial utilization [11]. The applications of TC are currently limited to fertilizers, insecticides, herbicides, fungicides, and coagulating agents in rubber industry. This study proposes microalgae as a better solution to tackle these problems.

Algal biorefineries are considered as an emerging solution for several global socio-ecological, economic, and environmental issues, thereby contributing to achieving UN sustainable development goals (SDG) [12]. Microalgae can contribute directly to nine SDGs and indirectly to all the 17 SDGs [13]. Photoautotrophic microalgae have garnered interest due to their small size, ability to utilize CO_2_, rapid growth rates, superior photosynthetic efficiency compared to plants, and lack of dependency on arable land for cultivation [14]. Microalgae can tolerate and valorize organic acids and phenolic compounds [15]. Chemical stress caused by these compounds often increase the secretion of extracellular polymeric substances (EPS). This offers an ideal valorization route for the microalgal production of EPS, which are industrially important compounds with applications as protective matrices, biosorbents, biolubricants and drag reducers, thickeners, preservatives, and phytoremediative agents [16,17]. Additionally, EPS from microalgae can be considered as high-value products due to their antioxidant, antiviral, anticoagulant, antitumor, and immune system-regulating properties [18,19]. However, the cost of production of microalgal EPS is higher by 10–100 times than plant and bacterial EPS. To compete with the market, microalgal EPS needs to focus on specific high-value applications such as prebiotic and antioxidant [20].

Despite its potential application as stressors for the production of EPS, the use of high amounts of organic acids and phenolic compounds derived from thermochemical processes in growth media has been known to inhibit the growth of microalgae [20]. In this study, we have integrated torrefaction with algal biorefineries to evaluate microalgal tolerance to TC and its valorization to produce EPS.

Among the approximately 200,000 known species of microalgae, *Chlamydomonas reinhardtii* was selected for this study, as it is an established model organism as well as a biological indicator for environmental pollution [21,22]. *C. reinhardtii* is well known for biodegradation, bioaccumulation, biotransformation, and enzymatic degradation of pesticides, herbicides, heavy metals, and phenolic pollutants [23,24]. It can grow in photoautotrophic, heterotrophic, and mixotrophic conditions. Under heterotrophic growth conditions, *C. reinhardtii* has been known to utilize acetic acid, and its stress tolerance, genetic modifications, biofuel production, and exopolysaccharide (EPS) production abilities are well-documented [25].

In this study, TC, a low-value torrefaction byproduct, was utilized as a nutrient additive for the cultivation of *C. reinhardtii*, and the effects of TC on microalgal growth and on EPS production and properties were evaluated. This is the first study to our knowledge that integrates the valorization of TC for the cultivation of C. *reinhardtii* along with quantitative and qualitative profiling of EPS in terms of yield, composition, and bioactivity. This study aims to integrate the concept of circular bioeconomy into the torrefaction process by utilizing TC to produce high-value EPS via algal biorefineries.

## 2. Results and Discussion

### 2.1. TC Yield

The torrefaction temperature significantly influences both the quantity and quality of the TC. The maximum TC yield was observed at 300 °C, while the minimum was observed at 225 °C (Table 1). This variation is attributed to the release of more condensable gases and organic acids at higher temperatures, resulting in a higher yield of condensate and lower pH values. These results are consistent with the previous studies on torrefaction of pine, wheat straw, and beech [26]. At lower temperatures, primarily moisture and low molecular weight volatiles are released, whereas higher temperatures facilitate the release of higher amount of volatile compounds and the decomposition of hemicellulose and cellulose [26,27].

### 2.2. TC Characterization

The GC-MS analysis of TC highlights the influence of temperature on its composition (Table 2). The analysis reveals 4, 13, 23, and 32 compounds at 225, 250, 275, and 300 °C, respectively, indicating that higher temperatures release a greater number of compounds [28]. All identified compounds in the TC samples were categorized as inorganic, esters, alcohols, diol, aldehydes, ketones, acids, alkanes, furans, phenols, pyridines, acetal, amine, thione, and benzene derivatives (Table 2). Across all temperatures, organic acids were the predominant compounds present in the TC. The high percentage of acetic acid and formic acid in TC results from the cleavage of acetoxy–methoxy groups present as side chains in the xylose of the xylan-containing hemicellulose fraction [29]. Hemicellulose degradation also led to furfurals formation.

Furfurals and 5-hydroxymethylfurfural (HMF) were quantified by HPLC. These compounds are the dehydration products of pentoses (furfural) and hexoses (HMF). Furfural and HMF act as important intermediates and precursors for the formation of acetic acid and formic acid via further reactions. The various factors that influence furfural release are temperature, reaction time, and acidity [30].

At lower temperatures (225 and 250 °C), furfural concentration was minimal (2.37 mg/mL), whereas it increased significantly at higher temperatures (6.97 mg/mL). However, further increase in temperature decomposes the furfural, resulting in the formation of other products such as acids and furan derivatives [31]. HMF exhibited a pattern similar to furfural. The yields of HMF and furfural vary according to the feedstock composition, process mode, temperature, residence time, and moisture content [30].

**Figure 1:**
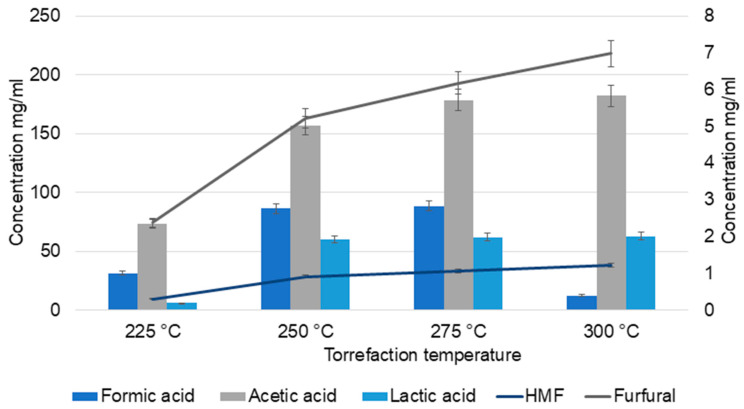
HPLC analysis of torrefaction condensate (TC).

HPLC analysis (Figure 1) of TC samples also confirmed the higher yield of acetic acid at higher temperatures. Acetic acid content increased from 73.4 mg/mL at 225 °C to 182 mg/mL (<2 times increase) at 300 °C. At the intermediate temperatures (250 °C and 275 °C), there was no significant change in the yield of acids compared to that at 225 °C. However, a gradual increase in formic acid content was observed at 225, 250, and 275 °C (31.3, 86.3, and 88.5, respectively), which then declined to 12.5 mg/mL at 300 °C. Similar patterns were observed in the catalytic wet torrefaction of wood cellulose pulp residue, which suggests the formation of other products by the conversion of formic acid at higher temperatures. The study also states that differences in process set up, time, and temperature of torrefaction result in variations in formic acid quantities, with a non-linear dependence on temperature [32].

Temperature also influenced lactic acid yields. Lactic acid and acetic acid are derived from erythrose and glycoaldehyde, formed by retro-aldol condensation of glucose [33]. At 225 °C, the lactic acid yield was 6 mg/mL and at all other temperatures, the yield was ≈60 mg/mL. In the case of pine wood torrefaction, at lower temperatures, the lactic acid yield was low; however, a gradual increase was observed in the percentage up to 250 °C [27]. No significant increase was observed in lactic acid formation at 250, 275, and 300 °C, which suggests the further degradation of lactic acid to acetaldehyde, CO, CO_2_, and small acids at higher temperatures [33].

In the case of CO_2_, a gradual decrease in concentration was observed with increasing temperature, as reported by Yajing He et al. [34]. At lower temperatures, decarboxylation reactions generate more CO_2_, whereas at higher temperatures, these reactions intensify, leading to increased production of CO [34]. The major compounds in the TC were primarily derived from the thermal decomposition of hemicellulose and lignin. Cellulose is more thermally stable, decomposes at higher temperatures, and contributes less significantly to the degradation products under the conditions studied. Thermal depolymerization of hemicellulose, lignin, and cellulose results in anhydrosugars, pyrans, and phenols, which subsequently decomposed into furans, alcohols, aldehydes, ketones, and acids [34].

### 2.3. Growth of C. reinhardtii

**Figure 2:**
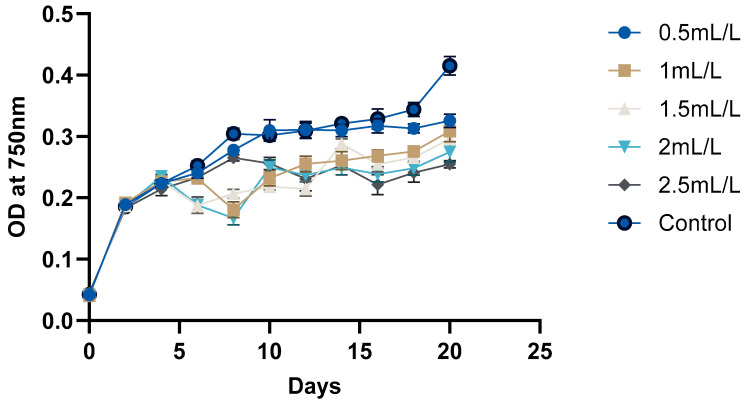
Growth analyses of *C. reinhardtii* based on OD at 750 nm.

**Figure 3:**
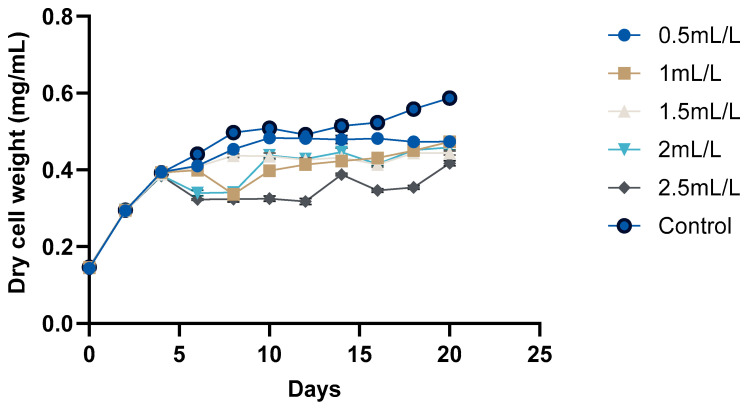
Growth analyses of *C. reinhardtii* based on DCW.

The growth curve of *C. reinhardtii* (Figure 2 and Figure 3) was monitored over 20 days to evaluate the impact of TC on its growth. According to the pH, GC-MS analysis, and HPLC analysis of the TC, TC at 225 °C was selected for the cultivation of *C. reinhardtii*. The curve revealed three distinct phases: a short lag phase, an exponential growth phase, and a stationary phase. Initial optical density (OD) at 750 nm of the cultures was approximately ≈0.041–0.045. During this phase, the cells adapted to the environment, gradually increasing in cell density. Using the same medium for pre-inoculum and 10% (*v*/*v*) inoculum facilitated faster adaptation, resulting in a short lag phase of one day. Next, the logarithmic phase was 4 days, (till day 6) during which the cells exhibited rapid growth. The OD values increased to 0.214–0.235, and the dry cell weight (DCW) reached 0.384–0.394 mg/mL. At this stage, different concentrations of TC were introduced to the media. After adding TC, pH of the medium decreased from 6.5 to 7 to around 4.5–5 depending on the concentration of TC. In the preliminary experiment, TC added from day 0 caused significant stress to the cells, particularly at higher concentrations, and cell survival was compromised. However, the addition of TC during the exponential growth phase showed a proportional decrease in microalgal growth with increasing TC concentration. For instance, at 2.5 mL/L TC, the DCW dropped from 0.384 to 0.323 mg which is a 26.7% decrease compared to the control (0.441), while at 2 mL/L TC, it decreased from 0.386 to 0.340 mg (a 22.9% decrease compared to control). These decreases in DCW are possibly due to the impact of acetic acid in the TC. In a previous study, acetic acid stress at pH 5 for 10 min caused an H_2_O_2_ burst and gradual degradation of DNA and photosynthetic pigments, indicating programmed cell death (PCD). Cells which underwent PCD released volatile organic compounds, which reduced the cell density [35]. However, lower concentrations (0.5 mL/L and 1 mL/L) showed minimal impact, indicating that cells could adapt and maintain growth under these conditions. After two days of TC addition, cells began to adapt, leading to a gradual increase in both OD and DCW. This adaptation highlights the resilience of *C. reinhardtii* in all the concentrations of TC. Mainly, the impact of acetic acid in TC could be considered here, because similar findings [25] in a previous study with acetic-acid-rich pyrolytic oil align with these results. The compositions of pyrolytic oil and TC are comparable; hence, the pattern observed in the growth of *C. reinhardtii* are also comparable. In the mentioned study, when 5% acetic acid was replaced by acetic-acid-rich pyrolytic oil, the cell growth was inhibited for 2 days, and on day 3, the cells caught up with the control cultures. However, when the concentration was increased to 10%, cell growth was significantly impacted and the cells could not recover [25]. Formic acid and furfurals in pyrolytic oil had limited impact on the growth of the *C. reinhardtii* due to their low concentration in the pyrolytic oil. In our study, the concentrations of formic acid and furfural in TC are lower than those in the pyrolytic oil to have a greater impact on the growth of the cells. After day 10, the cells reached stationary phase, where the nutrient-limiting factors are in balance and cell division is slowed down. This resulted in a stable OD value and DCW. Still, due to the evaporation and replenishment of media, minor differences were observed in the values.

### 2.4. Biomass and EPS Yield

The biomass yield followed the growth curve pattern, and the maximum biomass value was observed with the control group (1.53 mg/mL). As the TC concentration increased from 0.5, 1, and 1.5 mL/L, the biomass yield decreased to 1.21, 1.18, and 1.10 mg/mL, respectively, whereas at 2 mL/L concentration, a slight increase was observed in biomass yield compared to 1.5 mL/L (1.12 mg/mL). At 2.5 mL/L, the biomass yield was 1.53 mg/mL. This trend shows the gradual adaptation of the cells to TC.

In the case of EPS, the maximum yield was observed in samples treated with 2.5 mL/L (177.67 mg/L) and 2 mL/L (160.50 mg/L) of TC. At lower TC concentrations, the yields were 93.42 mg/L (0.5 mL/L), 104.92 mg/L (1 mL/L), 135.42 mg/L (1.5 mL/L), and 111.92 mg/L (control). No correlation was observed between the biomass concentration and EPS yield. At higher TC concentrations, the cells influenced by acetic acid stress caused increased EPS secretion [25]. At lower concentrations, the cells were utilizing the acetic acid (as in TAP medium, derived from observed trends), which favoured cell growth and explains the decrease in EPS compared to the control group. These patterns are consistent because in unfavourable conditions, *Chlamydomonas* cells enter a temporary stage called palmelloid, and in this stage, multiple changes occur including increased secretion of EPS [36]. Similar patterns have been observed previously in EPS production (proteins and polysaccharides) by *Chlamydomonas* sp. under Cd, Cu, Pb, and Zn stress [26] and in *C. reinhardtii* due to silver nanoparticles (Ag-NPs) [37].

### 2.5. Biochemical Analyses of Biomass

#### 2.5.1. Total and Free Amino Acid Analysis

**Figure 4:**
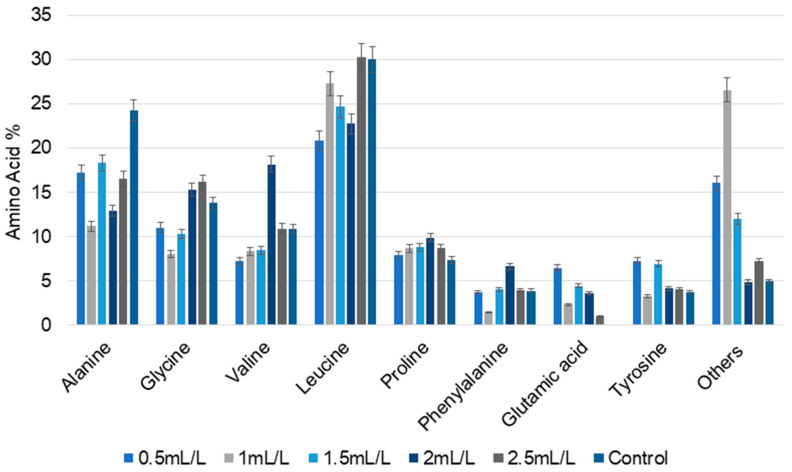
The percentage of total amino acid composition of *C. reinhardtii* cultivated in different concentrations of TC. (Data are presented as means of three replicates).

Amino acid composition is an important determinant of the nutritional content of microalgal biomass. Total and free amino acid composition were determined to assess the suitability of the treated biomass for use as nutraceuticals. The major total amino acids detected in the microalgal biomass are displayed in Figure 4. The main amino acids present in the all the samples were alanine, glycine, valine, leucine, proline, phenylalanine, and tyrosine. Glutamic acid and a few other amino acids were absent in the control, but observed in all other biomass treated with TC. Alanine content was lower at all the concentrations of TC compared to the control (24.23%). However, the amount of glycine progressively increased with increase in the concentrations of TC, with the exception of a slight difference at 1 mL/L (8.01%). For valine, the maximum value was observed at 2 mL/L (18.17%). Compared to the control, amino acid percentage in different concentrations of TC varied nonlinearly among the samples, showing that the TC concentration influenced the amino acid composition. Accumulation of branched chain amino acids indicates a shift towards stress-related pathways for increased protein biosynthesis.

In the case of free amino acids analysis, in the control, the major amino acid observed was only cystine (75%). The content of all other detected amino acids was less than 10%. In sharp contrast, more free amino acids were present in *C. reinhardtii* cultivated with TC (Table 3). These free amino acid profiles further support the influence of TC concentration on the amino acid composition.

#### 2.5.2. Lipid and Fatty Acid Analysis

**Figure 5:**
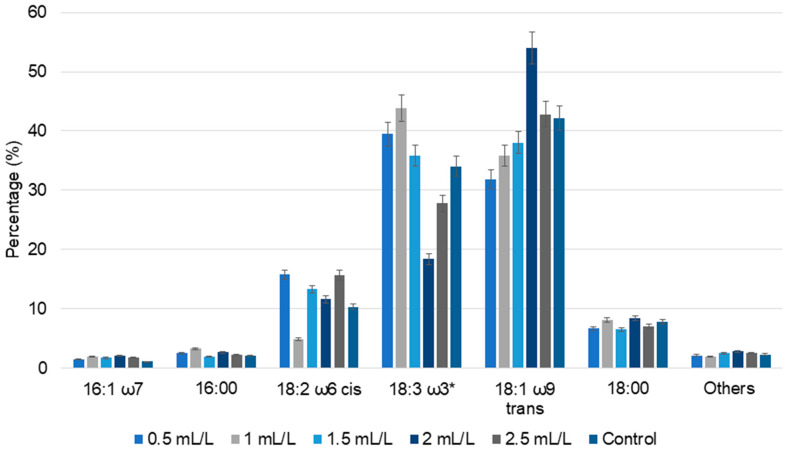
The percentage of fatty acid composition of *C. reinhardtii* cultivated in different concentrations of TC. 16:1 ω7—palmitoleic acid, 16:00—palmitic acid, 18:2 ω6 cis—linoleic acid, 18:3 ω3*—sum of oleic acid, linoelaidic acid, linolenic acid (which could not be separated by the used GC method), 18:1 ω9 trans—elaidic acid, 18:00—steric acid and others—all the minor detected fatty acids (Data are presented as means of three replicates).

The total lipid content in dried *C. reinhardtii* biomass is shown in Figure 5. The total lipid yields of biomass treated with 0.5 and 1 mL/L TC were 15.47% and 15.48%, respectively, which are slightly lower than that of the control (16.64%). These results are comparable with the total fatty acid content in *C. reinhardtii* ST21 grown in 4% SF5 pyrolytic oil, which was 9.75 ± 0.32%. The control group in this study displayed 20.01 ± 0.55% lipid content [20]. In a similar study, a wildtype *C. reinhardtii* strain grown in activated-carbon-treated SF5 pyrolytic oil at 10% acetic acid replacement had 13.04 ± 0.23% total fatty acid content, compared to 18.35 ± 0.14% in the control group [25]. In those studies, the amount of pyrolytic oil used was higher (4% and 10%) than the TC concentrations in our study and the impact of the pyrolytic oil on lipid production was much higher.

In case of TC, acetic acid is the major compound present. A comparison can be made with tris-acetate-phosphate (TAP) medium commonly used for heterotrophic microalgal cultivation, wherein acetic acid is the sole carbon source. At pH 7 and 17.4 mM acetic acid, the TAP medium supports vigorous growth of cells in the dark [35]. Based on this, lower TC concentrations in our study could promote cell growth and reduce stress, yielding a lower amount of total lipids compared to the control. Increasing TC concentration led to an increase in lipid percentage. The highest lipid concentration was observed for biomass treated with 2 mL/L TC (17.70%), closely followed by biomass treated with 2.5 mL/L TC (17.57%). In the case of 1.5 mL/L TC, the lipid percentage was 16.77%. These results show that higher TC concentrations induce greater stress responses in the cells, enhancing the lipid accumulation. Various studies have reported that nitrogen and sulfur starvation responses led to increased lipid accumulation in *C. reinhardtti* [38,39]. Similar trends were observed when *Chlorella vulgaris* NIES 227 was grown on hydrothermal liquefaction aqueous phase; initially reduced lipid accumulation was observed, followed by adaptive laboratory evolution of cells and improved tolerance.

In fatty acid analysis, elaidic acid, oleic acid, linoelaidic acid, linolenic acid, linoleic acid, steric acid, palmitic acid, and palmitoleic acid were found in all the biomass samples. Compared to the control, an increase in fatty acid percentage was observed in all the TC-treated *C. reinhardtii* biomass. Elaidic acid content increased with increasing TC concentrations up to 2 mL/L, and exhibited a slight decline at 2.5 mL/L. However, the total content of oleic acid, linoelaidic acid, linolenic acid (which could not be separated by the used GC method) displayed a slightly different pattern, with 39.51% and 43.84% in the lower concentrations of TC (0.5 and 1 mL/L) and 18.38% and 27.78%, respectively, in the higher concentrations of TC (2 and 2.5 mL/L). Polyunsaturated fatty acids such as linolenic acid showed alternatively increasing and decreasing patterns. Similar trends in fatty acid composition were observed in the cultivation of *C. reinhardtii* with pyrolytic oil [20,25]. Overall, these results indicate that the TC concentration regulates the lipid metabolism; even though the percentage of lipids can be higher in some controls, the fatty acid composition varies significantly with TC concentrations.

### 2.6. Structural Characterization of the Exopolysaccharides

#### 2.6.1. Molecular Weight of EPS

The weight average molecular weights (Mw) of the EPS samples treated with TC and control were as follows. In control, the Mw observed was 288 kDA, whereas the maximum Mw observed in 0.5 and 1.5 mL/L of TC treatment were 345 kDa and 350 kDa, respectively. The lowest Mw of 85.45 kDa was observed in 2 mL/L EPS, whereas 1.5 and 2.5 mL/L TC treatment resulted in Mw of 253 kDa and 232 kDa, respectively. The molecular weight of the purified EPS reported by Bafana [18] was 225 kDa from *C. reinhardtii* RAC. There are multiple factors that affect the molecular weight of EPS. Previous studies have reported that nitrogen stress, culture conditions such as light, pH, temperature, and mixing impacts the production and the Mw of EPS [16]. Another study about the role of structural features of EPS from different microalgae that used high pressure/solid acid catalyzed hydrolysis for depolymerization stated that the Mw of the EPS reduced with acid catalyzed hydrolysis [40].

#### 2.6.2. Fourier Transform Infrared (FTIR) Analysis

**Figure 6:**
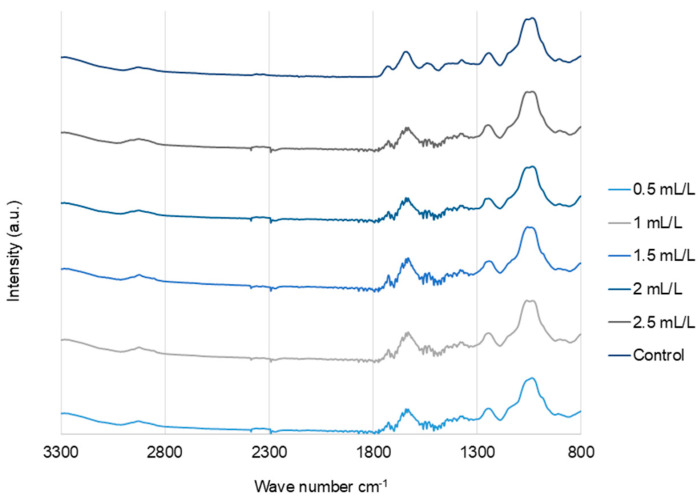
FTIR spectra of EPS from *C. reinhardtii* cultivated in different concentrations of TC in the medium.

The FTIR spectra (Figure 6) of the EPS from *C. reinhardtii* treated with and without TC were analyzed, and the results were very similar, indicating there was no significant effect of TC on the EPS functional groups. The major bands observed in all the samples were as follows. Bands around 3280–3292 and 2924–2932 cm^−1^ represent the O-H stretching vibrations and symmetric/asymmetric vibrations of C-H groups attributed to protein side chains and lipids, respectively [41]. Bands around 1724–1729 cm^−1^ were associated with O-acetyl groups. The bands at 1630–1637 cm^−1^ correspond to C=O stretching (amide I) and those at 1534–1541 cm^−1^ correspond to amide II (N-H bending/C-N stretching). Phosphate/sulphated regions were observed around 1230–1245 cm^−1^. The stretching vibrations of pyranose rings C-O-C, C-OH, and C-C in carbohydrates were observed between 1023 and 1055 cm^−1^ [18].

### 2.7. Biochemical Analysis of EPS

For the biochemical characterization of EPS, total sugars, neutral sugars, uronic acids, and proteins were evaluated using colorimetric assays (Table 4). The biochemical composition of the EPS was significantly altered by TC treatment concentration. While the neutral sugars remained stable (83.70–85.94%), total carbohydrates declined from 91.68% in the control to 74.25% at the highest dose. Conversely, uronic acid and protein contents increased, the latter rising substantially from 5.89% to 11.82%. This shift in composition suggests that TC treatment influences the structural and functional properties of EPS by enhancing its protein and acidic sugar components, while reducing overall carbohydrates. These results match with the studies on the toxicity effects of Ag-NPs on *C. reinhardtii*, where Ag-NPs stress caused an increase in EPS protein percentages [37].

### 2.8. Antioxidant Capacity of EPS

**Figure 7:**
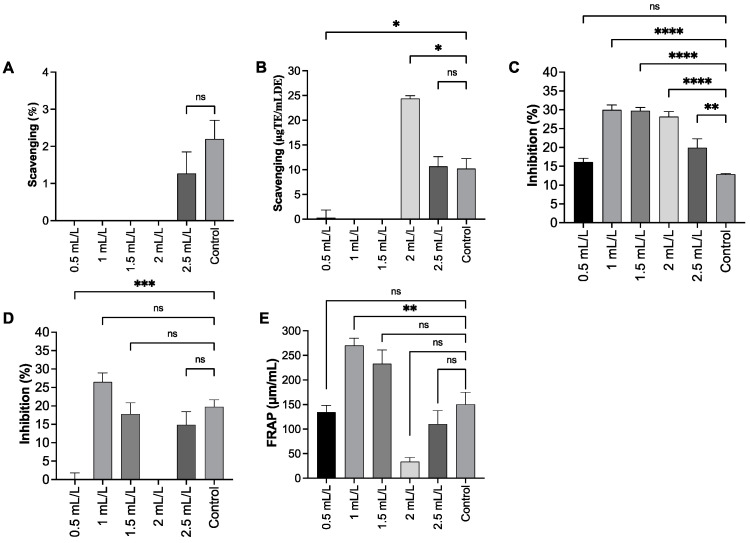
Antioxidant activity of EPS. (**A**). DPPH, (**B**). ABTS, (**C**). SOD, (**D**). OH radical assay, (**E**). FRAP. All experimental data are expressed as mean ± Standard Error of the Mean (*n* = 8); data are compared to the values of the control group. (*) indicates a statistically significant difference using ANOVA, followed by Dunnett’s multiple comparisons test: **** *p* < 0.0001, *** *p* < 0.001, ** *p* < 0.01, * *p* < 0.05 and ns—not significant (*p* > 0.05).

The antioxidant capacity of the extract was evaluated across the TC concentration gradient (0.5–2.5 mL/L) using 2,2-diphenyl-1-(2,4,6-trinitrophenyl) hydrazin-1-yl (DPPH), 2,2Hdiphenyl-1(3-ethylbenzothiazoline-6-sulfonic acid (ABTS) radical cation scavenging, superoxide dismutase (SOD)-like activity, hydroxyl radical (OH), and ferric reducing antioxidant power (FRAP) assays. The results indicated a variable and assay-dependent response. In the DPPH assay, the EPS did not show significant radical scavenging activity at any tested concentrations (0.5–2.5 mL/L). Although a slight increase was observed at 2.5 mL/L, this difference was not significant, compared with the control group (2.20 ± 0.50; *p* = 0.4931). Overall, the experiment confirms that the extract does not possess any significant antioxidant activity in the DPPH assay, even at the highest concentration tested. According to previous studies, EPS containing higher levels of phenolic compounds have been reported to exhibit higher DPPH radical scavenging activity [42]. The low DPPH activity indicated in this study suggests that the EPS contained a lower amount of phenolic compounds and was of high purity. In the ABTS assay, a significant increase in radical scavenging was detected specifically at the 2 mL/L concentration (24.39 ± 0.57%, *p* = 0.0216) compared to the control (10.20 ± 2.05%). The other concentrations either showed significantly less activity or were not statistically different. The most consistent effects were observed in the SOD-like assay. Treatment with the extract resulted in a statistically significant increase in activity at 1 mL/L (29.98 ± 1.31%, *p* < 0.0001), 1.5 mL/L (29.78 ± 0.84%, *p* < 0.0001), 2 mL/L (28.20 ± 1.37%, *p* < 0.0001), and 2.5 mL/L (19.91 ± 2.37%, *p* < 0.01) compared to the control (12.88 ± 0.20%). In case of sulfated polysaccharides of *C. reinhardtii* at a concentration of 0.01–1 mg/mL, a similar range of hydroxyl radical scavenging activity (22.29–80.9%) and a DPPH radical scavenging activity of 38–77% have been reported. Similarly, ABTS radical scavenging activity (9.8–81%), ferrous chelating ability (34.5–67.6%), and total antioxidant capacity (11.62–75%) are also reported [36]. The total antioxidant activity of EPS from *C. reinhardtii* expressed as 5.7 μg ascorbic acid equivalent/mg EPS and reducing power of 2.3 μg ascorbic acid equivalent/mg EPS was reported by Bafana A [32]. These results support the fact that the antioxidant activity of exopolysaccharides from this microalgae remains quite low [43] (Figure 7).

## 3. Materials and Methods

**Figure 8:**
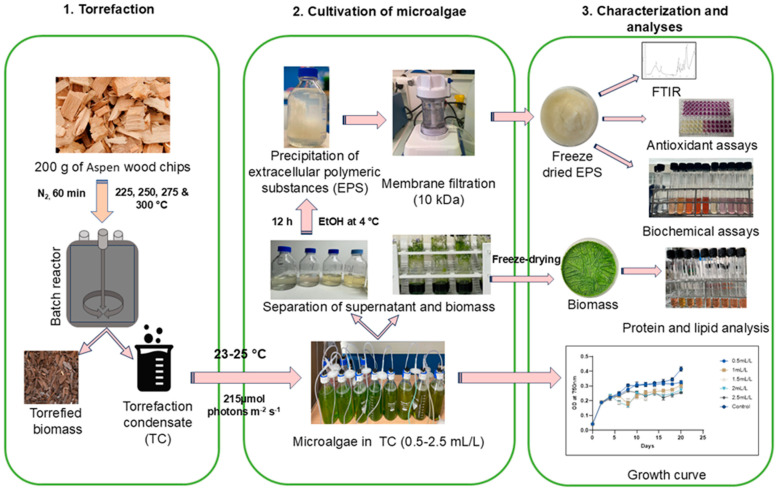
Diagram illustrating the experimental design. 1. Torrefaction, 2. cultivation of microalgae, 3. characterization and analyses of EPS and biomass of *Chlamydomonas reinhardtii*.

The overall experimental plan can be divided into three sections as torrefaction, cultivation of microalgae, and characterization and analyses, as illustrated in Figure 8.

### 3.1. Torrefaction of Biomass

Aspen wood chips (200 g) were torrefied at 225, 250, 275, and 300 °C at a residence time of 1 h in a batch reactor. To maintain an inert atmosphere, nitrogen gas was purged at a flow rate of 5 L/min. Torrefaction condensate (TC) produced by the release of the volatiles during the process was collected using a water-cooled condenser attached to the batch reactor in a glass bottle. TC yields at different temperatures were measured and recorded [44].

### 3.2. Characterization of TC

#### 3.2.1. GC-MS Analysis of TC

Composition analysis of the TC was performed by GC-MS as described in Doddapaneni et al. [39]. Due to the high water content in the TC, GC-MS samples were prepared by mixing the TC (0.6 mL) with methanol (0.4 mL). The mixtures were filtered with centrifuge filters (Thermo Scientific, Waltham, MA, USA, PTFE, 0.2 µm) and transferred to GC vials. Analyses were performed on an Agilent GC-MS (7890B) equipped with a MS detector Agilent 5977A and column HP-5MS–Ultra Inert (30 m, 0.25 mm ID, 0.25 µm film thickness; Agilent, Santa Clara, CA, USA). The oven was heated up to 200 °C at the rate of 2 °C/min and then to a final temperature of 280 °C, at a rate of 5 °C/min. The injection volume was 0.2 µL and the split ratio 20:1 [34].

#### 3.2.2. HPLC Analysis of TC

TC was diluted with milli-Q water (1:10, *v*/*v*) and centrifuged at 10,000 rpm for 5 min to remove the suspended solids. The supernatant was filtered with centrifuge filters (PTFE, 0.2 µm, Thermo Scientific, Waltham, MA, USA) and transferred into HPLC vials. HPLC system (Shimadzu Prominence-i LC-2030 3D Plus (Kyoto, Japan), with a Refractive Index Detector (RID) along with Photodiode Array Detector (PDA)) was used for the sample analysis. HPLC was performed using a Phenomenex Rezex ROA organic acid column (Torrance, CA, USA, 300 × 7.8 mm). The mobile phase was 0.005 M sulphuric acid at a flow rate of 0.5 mL/min. The oven temperature was maintained at 50 °C. The sample injection volume was 10 µL. Acids in the samples were detected using RID, while HMF and furfurals in the sample were detected using a PDA detector at 283 nm [45].

### 3.3. Cultivation of Microalgae

*C. reinhardtii* (11-32a) strain was purchased from Culture Collection of Algae at Goettingen University (SAG), Göttingen, Germany (http://www.epsag.uni-goettingen.de/ (accessed on 20 January 2021)). The culture medium used was Sueoka’s High salt medium (HS/HSM). It was prepared by mixing 5 mL of salt solution, 5 mL phosphate solution, and 1 mL of Hutner’s trace elements solution and making up the volume to 1 L with distilled water, as described by Sueoka et al. [46], and sterilized by autoclaving at 121 °C for 15 min. The experiments were conducted in 450 mL of media in 500 mL Schott glass bottles. Starter cultures were maintained according to the instructions of the culture collection, and 10% (*v*/*v*) of starter culture/ inoculum with an OD of ≈0.2–0.3 was used to inoculate the bottles. Continuous artificial illumination and aeration was provided, with irradiance up to 215 µmol photons m^−2^ s^−1^ and air flow rate of 1 mL/ min. The bottles were maintained at room temperature (23–25 °C). OD of the cultures was monitored every 48 h, and fresh medium was added to compensate for losses. After 6 days, when the cultures reached an OD of ≈0.1–0.2, TC was added to the culture medium in different concentrations such as 100, 250, 500, 750, and 1000 µL/L and the cultures were grown for 20–22 days. The pH of the medium after the addition of TC was noticed using pH papers. Cell growth was measured by OD and DCW method. The OD was measured at 750 nm using a UV/Vis spectrophotometer. For DCW, 10 mL of cells were collected by vacuum filtration on pre-dried and pre-weighed Whatman filter paper, dried at 120 °C overnight and re-weighed, until the weight was stable. All experiments were conducted in triplicate and a control culture was grown in medium without TC.

### 3.4. Recovery of Biomass and Extraction of EPS

The culture was centrifuged at 10,000 rpm for 5 min at 20 °C to separate the biomass from the medium. The separated biomass was freeze-dried and stored at −20 °C for further analysis. EPS in the supernatant was precipitated by alcohol precipitation. The supernatant was mixed with ice-cold ethanol (98%) in a 1:4 ratio and incubated overnight at 4 °C. After incubation, the EPS were recovered by centrifuging at 10,000 rpm for 3 min at 4 °C. The resulting pellets were collected and resuspended in milli-Q water. The EPS solutions were further purified using an Amicon filter cell (10 kDa MWCO polyethersulphone membrane (Biomax^®^ Ultrafiltration discs, Millipore, Burlington, MA, USA) until the conductivity of the sample reduced to 0.2 mS/m. Purified samples were freeze-dried and stored for further studies [47].

### 3.5. Biochemical Analyses of C. reinhardtii

#### 3.5.1. Protein and Amino Acid Analysis

The free and total amino acid contents in freeze-dried microalgal biomass were determined by GC-MS. For the determination of total amino acids, 0.02 g of the sample were hydrolysed at 120 °C for 15 h with 2 mL of 6 M HCL. After hydrolysis, the samples were kept under a stream of nitrogen for drying at 95 °C. The samples were dissolved in 2 mL milli-Q water and used for GC-MS analysis. For the determination of free amino acids in the freeze-dried biomass, 0.05 g of the biomass was added to 1.5 mL of 0.1 M HCl and shaken vigorously at 1400 rpm for 5 min at room temperature. The samples were then centrifuged at 21,000× *g* for 15 min at 4 °C. The supernatant was collected and stored at −80 °C until analysis. The same sample preparation steps were followed for both total and free amino acid determination by GC-MS.

An amount of 100 µL of amino acid solutions were mixed with 250 µL of acetonitrile and the mixture was centrifuged at 21,000× *g* for 3 min. After centrifugation, the supernatant was collected. A total of 100 µL of the supernatant was mixed with 100 µL of internal standard solution of 5 µg/mL DL-norleucine in a heat-resistant, capped Eppendorf tube. The samples were evaporated under a stream of nitrogen, mixed with 50 µL of dichloromethane by gently vortexing, and evaporated again under the stream of nitrogen. After drying, the samples were mixed well with 100 μL of MTBSTFA (Supelco 77626, Sigma-Aldrich, St. Louis, MO, USA) and 100 μL of acetonitrile then incubated at 100 °C for 1 h for derivatization. After the incubation, the tubes were centrifuged at 21,000× *g* for 15 min at 4 °C and 200 µL supernatant was transferred to a sample vial. The sample vials were centrifuged at 2000 rpm for 5 min before the GC-MS analysis.

Amino acids were analyzed using Shimadzu GCMS-QP2010 Ultra gas chromatograph system equipped with a mass detector (MS, Kyoto, Japan). Phenomenex Zebron ZB-5MS silicon-filled capillary column (30 m × 0.25 mm, 0.25 μm layer thickness) and carrier gas helium with a flow rate of 1 mL/min were used for the separation. The detector (MS) was maintained at a temperature of 325 °C and the ion source at 300 °C. The sample injector was operated by a 2 mm diameter straight liner at 280 °C. The sample injection volume was 0.5 µL in split mode (distribution flow 100), and the scans ranged from *m*/*z* = 25 to 500. During the analysis phase, the column was maintained at 100 °C for 2 min, then heated to 298 °C at a rate of 5 °C/min, and held for 25 min. Quantification was performed using analytical standards (Supelco A6407, A6282) [48].

#### 3.5.2. Lipid and Fatty Acid Analysis

Lipid extraction of the lyophilised microalgal biomass was performed using a chloroform/methanol (2:1, *v*/*v*) solvent system. In a glass screw cap tube, 0.3 g of the biomass was added to 1 mL of methanol and 2 mL chloroform, mixed vigorously for 90 s and incubated for 30 min at 40 °C in a water bath. Following the incubation, 1.25 mL of 2% NaCl solution and 1.25 mL of chloroform were added and mixed well. The mixture was centrifuged at 1700× *g* for 20 min, and the chloroform layer was transferred to pre-weighed glass tubes using a glass Pasteur pipette. The transferred solvents were dried under a stream of nitrogen, and the tubes were re-weighed to calculate the percentage of lipids in microalgal biomass.

After weighing, 1.5 mL of 5% sulphuric acid solution in methanol were added to the tubes containing the dried samples for fatty acid methyl ester (FAME) analysis. The solution was mixed well and incubated at 50 °C for 1 h with 30 s of mixing every 15 min. After incubation, the tubes were placed in ice-cold water to cool down and later, 1 mL of milli-Q water and 1.5 mL of hexane were added to the tubes. The tubes were mixed vigorously and allowed to form layers. The upper layers were transferred to HPLC vials and dried under stream of nitrogen. Then, 500 µL of hexane were added, and the vials were stored at −20 °C until GC analysis.

The GC-MS analysis was performed similar to that in Section 3.5.1, with some differences. The flow rate of the helium carrier gas was 30 cm/s. The sample injection volume was 1 µL in split mode (distribution flow 100). During the analysis, the column temperature was raised from 160 °C to 260 °C at a rate of 2.5 °C/min, then heated at a rate of 5 °C/min to 298 °C and held for 15 min. Fatty acids were identified using PUFA-2 (Sigma 47015 U, Sigma-Aldrich) and 38 FAME Mix (Supelco CRM47885, Sigma-Aldrich) as reference standards [48].

### 3.6. Structural Characterization of EPS

#### 3.6.1. Size-Exclusion Chromatography of EPS

EPS solution with a concentration of 0.5 mg/mL was prepared in 0.1 M NaNO_3_ solution, and the EPS were completely dissolved by heating in a boiled water bath with vigorous stirring. The hot (~60 °C) samples were filtered through a 0.22 μm cellulose acetate syringe filter (25 mm diameter), then transferred to HPLC vials, and cooled to 40 °C. Size- exclusion chromatography of the samples was performed using a Shimadzu liquid chromatograph (Nexera X2 LC-30AD with CBM-20A controller, Kyoto, Japan). Two Shodex OHpak SB-806MHQ, Kyoto, Japan (300 × 8 mm) columns in series connected with OHpak SB-G guard column were used for the separation. The mobile phase was 0.1 M NaNO_3_ with a flow rate of 0.8 mL/min, and the CTO-20 AC column oven temperature was maintained at 60 °C. In total, 100 µL of the samples were injected to the Nexera X2 SIL-30 AC autosampler at 40 °C. The total running time of one sample was 45 min, and samples were detected using RID-10A refractive index detector and analytical data system LabSolutions. A standard curve of 12 pullulan standards ranging from 0.342 to 2400 kDa was used to estimate the molecular weight of the samples using LabSolutions software version 5.97 (Shimadzu, Kyoto, Japan) [48].

#### 3.6.2. Fourier Transform Infrared (FTIR) Analysis

FTIR analysis of dried EPS samples was performed using FTIR (iS50) Nicolet (Thermo Fisher Scientific, Waltham, MA, USA). Samples were dispersed on an attenuated total reflectance diamond crystal, and infrared spectra were recorded at room temperature in absorption mode between 400 and 4000 cm^−1^. Data analysis was performed using Omnic software version 9.2 [49].

### 3.7. Biochemical Analyses of EPS

All analyses were performed in duplicate for the standards and in triplicate for the samples.

#### 3.7.1. Total Sugar Estimation

EPS solutions of 1 mg/mL were prepared in milli-Q water for the biochemical analysis. The total carbohydrate concentration was determined by the Dubois phenol–sulfuric acid method. An aliquot of 500 μL EPS solution was mixed with 500 μL of phenol solution (50 g/L in water) in a screw cap glass tube. Then, 2.5 mL of concentrated H_2_SO_4_ was added immediately. The mixture was incubated for 10 min at room temperature and vortexed at 3000 rpm for 10 s. Again, it was incubated at room temperature for 15 min and then at 35 °C in a water bath for 30 min. A UV-Vis spectrophotometer (V-630 Jacso, Nantes, France) was used to measure the absorbance at 483 nm of the solutions. The standard curve was prepared using 0.1 g/L glucose solution and milli-Q water was used as blank [50].

#### 3.7.2. Estimation of Uronic Acid and Neutral Sugar

Uronic acid and neutral sugar content in the purified EPS samples were evaluated using meta-hydroxyldiphenyl (m-HDP) and resorcinol, respectively, using glucose and glucuronic acid as standards. For the estimation of uronic acids, 200 μL of EPS solution (1 mg/mL) was added to 1 mL of Borax solution (0.12 M sodium tetraborate in concentrated H_2_SO_4_) in a screw cap glass tube, mixed well by vortexing, and incubated at 90 °C in a water bath for 1 h. After the incubation, 200 μL of freshly prepared m-HDP solution (100 mg in 1 mL DMSO) was instantly added and vortexed. The tube was then incubated exactly for 2 min at 90 °C in a water bath, and the absorbance was measured at 520 nm using a UV-Vis spectrophotometer (V-630 Jacso, France) [51].

For neutral sugar estimation, 200 μL of EPS solution (1 mg/mL) was added to 200 μL of resorcinol and 1 mL of H_2_SO_4_ (80%) in a screw cap glass tube and vortexed. Then, the tubes were incubated for 30 min at 90 °C in a water bath, followed by incubation in the dark at room temperature. After the incubation, the solution was diluted with 1.4 mL of milli-Q water and vortexed thoroughly. Absorbance at 450 nm was measured using a UV-Vis spectrophotometer (V-630 Jacso, France). Calculation of neutral sugars was performed, and the final results were expressed in mg/g of d-glucose equivalent. For uronic acids, the final results were expressed as mg/g d-glucuronic acid equivalent [52].

#### 3.7.3. Protein Estimation

Protein concentration in the EPS was determined by the Bradford method (Coomassie Brilliant Blue G-250 method). Bovine serum albumin (BSA) was used as the standard and milli-Q water was used as blank. A volume of 200 μL of Bradford reagent (Bio-Rad, Hercules, CA, USA) was added to 200 μL of EPS solution (1 mg/mL) and 600 μL milli-Q water. The mixture was vortexed and the tubes were incubated at room temperature for 10 min. After the incubation, the absorbance was measured at 595 nm using a UV-Vis spectrophotometer (V-630 Jacso, France) [53].

### 3.8. Determination of Antioxidant Capacity of EPS

#### 3.8.1. DPPH Radical Scavenging Activity Assay

DPPH radical scavenging activity of the purified EPS (0.1% solution) was evaluated according to the method of Brand–Wereiams, as modified by Miliauskas. A total of 20 μL of EPS solution was mixed with 180 μL of 0.1 mM DPPH in absolute ethanol and incubated in the dark for 30 min at room temperature. Trolox solution (1 mM) prepared in absolute ethanol was used as standard, and milli-Q water was used as blank. After incubation, the absorbance was measured at 570 nm using an OPTIMA microplate reader (FLUO star, Cary, NC, USA).

#### 3.8.2. ABTS Free Radical Scavenging Activity Assay

ABTS assay reagents, standard solutions of ascorbic acid, and Trolox were prepared as described by Premarathna et al., 2024 [49]. A total of 120 μL of ABTS working solution (3.5 mM ABTS + 1.225 mM potassium persulfate) was mixed with 30 μL of EPS solution (0.1%) and incubated in the dark for 30 min at 37 °C. Trolox and ascorbic acid were used as standards, and milli-Q water was used as blank. The absorbance of the colour developed was measured at 734 nm using a microplate reader [54].

#### 3.8.3. SOD Scavenging Activity Assay

SOD scavenging activity of the purified EPS solution (0.1% *w*/*v*) was assessed using the modified procedure described by Li et al. (2012) [55]. EPS solution (12 µL) was mixed with 50 mM Tris-HCl buffer (pH 8) containing 10 mM EDTA (180 μL) and 60 mM pyrogallol (2 μL). The mixture was incubated at 37 °C for 5 min, and its absorbance was measured at 325 nm using a microplate reader. L-ascorbic acid and milli-Q water were used as the positive control and blank, respectively. Percentage inhibition of superoxide radical was calculated using the formula:

$$SOD\text{-}like\;scavenging\;inhibition\left(\%\right)=\left(\frac{AB-AE}{AB}\right)\;*\;100$$

where AB is the absorbance of the blank and AE is the absorbance of the EPS sample [55].

#### 3.8.4. Hydroxyl Radical Scavenging Activity

The hydroxyl radical (OH) scavenging activity of the purified EPS samples was determined by mixing 20 μL of EPS solution (0.1%) with 45 μL of H_2_O_2_ (6 mM), 20 μL of sodium salicylate (20 mM), and 65 μL of FeSO_4_·7H_2_O (1.5 mM). The mixture was incubated in the dark at room temperature (22–25 °C) for 30 min and then the absorbance was measured at 562 nm using a microplate reader. Trolox was used as the positive control and milli-Q water used as blank. Hydroxyl scavenging capacity in percentage was calculated as follows:

$$OH\;radical\;scavenging\;capacity\;(\%)=\left(\frac{AB-AE}{AB}\right)\;*\;100$$

where (AB) absorbance of the blank and (AE) absorbance of the sample (EPS solutions) [54].

#### 3.8.5. FRAP Assay

FRAP assay of the EPS samples were performed according to the protocol described by Premarathna et al., 2024 [49]. FRAP reagent was prepared by mixing 10 mM TPTZ (2,4,6-tri(2-pyridyl)-s-triazine) in 40 mN HCl, 300 mM Na acetate buffer (3.6 pH), and 20 mM FeCl_3_·6H_2_O in 10:1:1 (*v*/*v*/*v*) ratio. Then, 180 μL of FRAP working solution was mixed with 6 μL of EPS solution (0.1%) and incubated in the dark for 30 min at 37 °C. Trolox was used as standard, and acetate buffer was used as blank. The absorbance of the colour developed was measured at 593 nm using a microplate reader. FRAP value was calculated as per the equation below and expressed in mmol Fe^2+/^g of sample.

$$FRAP\;value=\left(\frac{A_{1}-A_{0}}{A_{C}-A_{0}}\right)\;*\;2$$

where A_1_ is the absorbance of the sample, A_0_ is the absorbance of the blank, and A_c_ is the absorbance of the positive control [56].

### 3.9. Statistical Analyses

For antioxidant capacity measurements, all statistical analyses were performed using GraphPad Prism version 10.0.1 for MacOS (San Diego, CA, USA), including graph generation. Statistical significance was defined as **** *p* < 0.0001, *** *p* < 0.001, ** *p* < 0.01, and * *p* < 0.05. One-way ANOVA was employed for multiple comparisons across experimental groups. Dunnett’s post hoc test was applied to assess significant differences between variables. All experiments were conducted in quadruplicate (*n* = 4). Asterisks (*) denote statistically significant differences determined by ANOVA followed by Dunnett’s multiple comparisons test.

All the other experiments were conducted in triplicate, and results are expressed as mean ± standard deviation.

## 4. Conclusions and Future Perspectives

The present study demonstrates the feasibility of using TC as a nutrient additive for cultivating the microalgae *C. reinhardtii* to enhance EPS production. TC contains myriads of compounds, and the temperature of torrefaction impacts its composition. A gradual adaptation of microalgal cells to TC was observed, with the highest biomass yield (1.53 mg/L) and EPS yield (177.67 mg/L) achieved at higher concentrations of TC. The chemical stress induced by the TC altered the biochemical composition of both the biomass and EPS. The presence of higher concentrations of free amino acids in the treated samples compared to the control, in addition to the presence of unsaturated fatty acids such as oleic acid, linolenic acid, and linoleic acid are promising initial steps in the direction of using TC in microalgae cultivation for dietary supplement applications. Indeed, these results open up opportunities in the field of biofuels, biomolecules, and nutraceuticals for the utilization of microalgal biomass and EPS.

This study illustrates the potential integration of thermochemical processes with an algal biorefinery for the production of a high-value product with unique characteristics. However, the absence of pH-matched controls constrains the interpretation of the effect of pH and chemical toxicity. Exploring the effect of TC generated in different temperatures, detoxification or neutralization of TC, and scalability of this process would establish it as a viable and sustainable method. This work also demonstrates the cascading use of a torrefaction byproduct, the TC, which is produced in already established commercial torrefaction companies to form a valorization route of low-value TC into high-value EPS, integrating the principles of circular bioeconomy.

## Figures and Tables

**Table 1:** Torrefaction of aspen biomass and TC yield in different temperatures.

No	Temperature (°C)	Biomass (g)	TC Yield (mL)	pH
1	225	172.23	17 ± 2	2.7
2	250	164.56	24 ± 2	2.4
3	275	134.24	28 ± 2	2.1
4	300	68	50 ± 5	2.2

**Table 2:** GC-MS analysis of TC (relative peak area distribution—area (%)).

No	Group ^1^	Composition of Torrefaction Condensate	GC-MS % Area			
		Compound	225 °C	250 °C	275 °C	300 °C
1	IO	Carbon dioxide	10.38	6.65	2.66	2.30
2	Es	Acetic acid, methyl ester	-	7.49	5.00	1.95
3	Ac	Acetic acid	41.86	35.95	30.82	29.99
4	K	1-hydroxy-2-butanone	-	-	3.09	-
5	K	1-hydroxy-2-propanone	-	6.72	6.95	7.75
6	Fu	Furfural	4.53	8.72	5.61	5.36
7	Ac	Benzoic acid	5.84	-	-	-
8	K	1-(2,4,6-trihydroxy-3-methylphenyl)-1-butanone	-	2.95	-	-
9	Es	2-butoxyethyl acetate	-	4.90	-	-
10	Am	*N*-methyl-1,3-Propanediamine	-	9.97	-	8.61
11	Di	1,2-ethanediol	-	2.75	-	0.68
12	Fu	2-furanmethanol	-	-	1.16	2.49
13	K	1-(acetyloxy)-2-propanone	-	-	1.05	1.18
14	Fu	(*S*)-2-(furan-2-yl)-2-methoxyethanol	-	6.25	3.60	-
15	Fu	tetrahydro-2,5-dimethoxy-furan	-	3.57	4.27	0.45
16	Ace	1,1-dimethoxy-heptane	-	1.30	1.22	-
17	Ph	Phenol	-	-	1.61	2.51
18	Ace	Hexanal dimethyl acetal	-	-	1.11	-
19	Alc	Cyclobutanol	-	-	13.21	-
20	Ph	2,6-dimethoxy-phenol	-	2.78	4.23	6.25
21	Ac	3,5-dimethoxy-4-hydroxyphenylacetc acid	-	-	4.22	4.56
22	Ph	1-(4-hydroxy-3,5-dimethxyphenyl)-ethanone	-	-	1.27	1.83
23	Ph	2,6-dimethoxy-4-(2-propenyl)-phenol	-	-	1.87	3.21
24	Ben	4-hydroxy-3,5-dimetoxy-benzaldehyde	-	-	1.21	0.96
25	Ben	4-ethylbiphenyl	-	-	1.92	-
26	Ph	5-tert-butylpyrogallol	-	-	1.29	-
27	Ad	Glyceraldehyde	-	-	0.31	-
28	Ace	Octanal dimethyl acetal	-	-	1.47	-
29	K	2,3-butanedione	-	-	-	0.22
30	Pyr	2,4-dihydroxypyridine	-	-	-	1.41
31	Th	*N*-methylthio-formamide	-	-	-	0.48
32	Fu	5-methyl-2-furancarboxaldehyde	-	-	-	-
33	Es	2-butoxy-1-methylethyl butanoate	-	-	-	0.86
34	K	3-methyl-1,2-cyclopentanedione	-	-	-	1.66
35	Ph	2-methoxy-phenol	-	-	-	0.95
36	Ph	Creosol	-	-	-	0.68
37	Ph	4-ethyl-2-methoxy-phenol	-	-	-	0.72
38	Ben	4-hydroxy-3-methoxy- benzoic acid	-	-	-	2.83
39	Ph	2-methoxy-4-(1-propenyl)-phenol	-	-	-	0.93
40	K	1-(2,6-dihydroxy-4-methoxyphenyl)-ethanone	-	-	-	2.34
41	Ph	Homovanillyl alcohol	-	-	-	0.75
42	Ben	*N*-butyl-benzenesulfonamide	-	-	-	2.05
43	Ac	Propanoic acid	-	-	-	0.28
44	K	4-hydroxy-2-butanone	-	-	-	2.49
45	K	2-pentanone	-	-	-	1.26

^1^ IO—inorganic, Es—esters, Alc—alcohols, Di—diol, Ad—aldehydes, K—ketones, Ac—acids, Fu—furans, Ph—phenols, Pyr—pyridines, Ace—acetal, Am—amine, Th—thione, Ben—benzene derivatives.

**Table 3:** The percentage as a mean value of free amino acid composition of *C. reinhardtii* cultivated in different concentrations of TC.

No	Name	Free Amino Acid %					
		0.5 mL/L	1 mL/L	1.5 mL/L	2 mL/L	2.5 mL/L	Control
1	Alanine	19.13	17.45	17.52	15.29	18.26	5.01
2	Glycine	5.79	5.75	5.39	4.59	3.64	1.60
3	Beta-Alanine	0.34	0.28	0.23	0.15	12.36	4.05
4	Valine	7.06	6.93	6.73	6.82	4.79	1.21
5	Leucine	21.92	21.86	21.00	22.41	8.95	2.65
6	Isoleucine	7.04	7.01	6.50	7.29	4.71	1.17
7	Proline	13.81	12.43	12.58	14.44	16.70	4.87
8	Methionine	0.88	1.40	1.49	1.70	0.59	0.20
9	Serine	3.63	3.73	3.08	3.28	1.32	0.90
10	Threonine	2.12	3.34	3.06	3.28	1.82	0.80
11	Phenylalanine	2.32	2.44	2.38	2.78	0.71	0.15
12	Aspartic acid	1.30	1.60	0.84	1.65	0.21	0.02
13	Cysteine	0.09	0.16	0.52	0.17	2.43	0.89
14	Glutamic acid	3.90	4.55	1.60	4.73	3.82	0.88
15	Lysine	2.21	1.54	0.79	1.10	1.11	0.07
16	Tyrosine	5.18	5.79	5.32	7.18	2.09	0.30
17	Tryptophan	0.99	2.09	2.03	0.00	1.36	0.18
18	Cystine	0.83	1.03	8.47	2.45	14.41	75.02

**Table 4:** Compositional analyses of EPS samples; carbohydrates, uronic acids, and proteins.

EPS Samples [% Mass (g/100 g EPS)]	Total Carbohydrate (%)	Neutral Sugars (%)	Uronic Acids (%)	Protein (%)
Control	91.68	85.42	14.58	5.89
0.5 mL/L	90.40	85.94	14.06	7.33
1 mL/L	78.32	83.70	16.30	9.49
1.5 mL/L	74.25	84.37	15.63	11.31
2 mL/L	80.55	83.76	16.24	7.21
2.5 mL/L	88.18	84.02	15.98	11.82

## Data Availability

The original contributions presented in this study are included in the article. Further inquiries can be directed to the corresponding author.

## Abbreviations

The following abbreviations are used in this manuscript:

TC	Torrefaction condensate
EPS	Exopolysaccharide
OD	Optical density
DCW	Dry cell weight
PCD	Programmed cell death
